# Evaluation of patient-reported severity of hand–foot syndrome under capecitabine using a Markov modeling approach

**DOI:** 10.1007/s00280-020-04128-7

**Published:** 2020-08-27

**Authors:** Eduard Schmulenson, Linda Krolop, Sven Simons, Susanne Ringsdorf, Yon-Dschun Ko, Ulrich Jaehde

**Affiliations:** 1 Department of Clinical Pharmacy, Institute of Pharmacy, University of Bonn, An der Immenburg 4, 53121 Bonn, Germany; 2 Department of Internal Medicine, Evangelische Kliniken Bonn gGmbH, Johanniter Hospital, Bonn, Germany

**Keywords:** Markov model, Capecitabine, Hand–foot syndrome, Patient-reported outcomes

## Abstract

**Purpose:**

The inclusion of the patient’s perspective has become increasingly important when reporting adverse events and may assist in management of toxicity. The relationship between drug exposure and toxicity can be quantified by combining Markov elements with pharmacometric models. A minimal continuous-time Markov model (mCTMM) was applied to patient-reported outcomes using hand–foot syndrome (HFS) induced by capecitabine anti-cancer therapy as an example.

**Methods:**

Patient-reported HFS grades over time of 150 patients from two observational studies treated with oral capecitabine were analyzed using a mCTMM approach. Grading of HFS severity was based on the Common Terminology Criteria for Adverse Events. The model was evaluated by visual predictive checks (VPC). Furthermore, a simulation study of the probability of HFS severity over time was performed in which the standard dosing regimen and dose adjustments according to HFS severity were investigated.

**Results:**

The VPC of the developed dose–toxicity model indicated an accurate description of HFS severity over time. Individual absolute daily dose was found to be a predictor for HFS. The simulation study demonstrated a reduction of severe HFS using the recommended dose adjustment strategy.

**Conclusion:**

A minimal continuous-time Markov model was developed based on patient-reported severity of hand–foot syndrome under capecitabine. Thus, a modeling framework for patient-reported outcomes was created which may assist in the optimization of dosage regimens and adjustment strategies aiming at minimizing symptom burden during anti-cancer drug therapy.

**Electronic supplementary material:**

The online version of this article (10.1007/s00280-020-04128-7) contains supplementary material, which is available to authorized users.

## Introduction

Anticancer treatment is frequently associated with adverse events. Thus, the management of toxic effects is a major aspect of a successful therapy. To account for the severity of adverse events, the Common Terminology Criteria for Adverse Events (CTCAE) are widely used for evaluation of toxicity [1]. The grading of adverse events is conducted by the study personnel. However, since reports suggest that this method is associated with underestimations of adverse event severity [2, 3], the patient’s perspective has become increasingly important. Therefore, a version of Patient-Reported Outcomes (PRO-CTCAE) has been developed and is increasingly used [4].

Pharmacokinetic–pharmacodynamic (PKPD) modeling approaches have proved to be useful to quantify the relationship between drug exposure and toxicity. Whereas some adverse events can be classified by metric data, such as myelosuppression [5], others, such as the severity of hand–foot syndrome or fatigue, lack objectively quantifiable parameters. Particularly, patient-reported data are often categorical as they are generated by subjective grading. One possibility to link categorical longitudinal toxicity data with drug exposure are Markov models. By applying these models, the probability of developing an adverse event of a certain grade can be estimated. Karlsson et al. introduced Markov models into the field of PKPD by analyzing sleep stages in insomnia patients [6]. Since then, Markov models were applied to a wide field of scenarios, such as diarrhea and rash, in cancer patients [7, 8], proteinuria [9] or improvement scores in rheumatoid arthritis [10].

Capecitabine is an orally administered prodrug of the cytotoxic agent fluorouracil (5-FU) used for the treatment of various tumor entities, such as colorectal and breast cancer. The metabolic activation of capecitabine to 5-FU occurs primarily in tumor cells minimizing the systemic toxic effects of 5-FU [11]. However, it causes a higher incidence of hand–foot syndrome (HFS) than intravenously administered 5-FU [12, 13]. Because the occurrence and severity of HFS were assumed to be dose-dependent the management of HFS toxicity includes dose reductions [14, 15]. Hénin et al. already linked capecitabine exposure to HFS toxicity using a Markov modeling approach [16] but could only consider CTCAE grades which were described by clinicians. Therefore, a model-based extension towards a patient perspective would allow to improve the management of adverse events.

The aim of this project was to develop a modeling and simulation framework to describe and predict patient-reported HFS severity in patients treated with capecitabine. Based on this example, the suitability of Markov models to simulate the time course of patient-reported toxic symptoms should be assessed.

## Methods

### Patients and data

For this work, raw data from a total of 150 capecitabine-naïve patients were pooled from two open, prospective multi-centered observational cohort studies. Both studies aimed at evaluating the effect of pharmaceutical care on adherence of capecitabine-treated patients and were approved by the ethics committee at the Faculty of Medicine of the University of Bonn [17, 18]. A summary of the observed data can be found in Table 1. Capecitabine was administered orally twice daily as an intermittent regimen in 3-week cycles (14 days of treatment and seven-day break). Dose modifications, treatment interruptions and discontinuations were conducted at the sole discretion of the treating oncologists.

**Table 1:** Summary of observed data [17, 18]

Patients analyzed (male/female)	150 (39/101)
Age (years), median (range)	62 (28–93)
Tumor entity	
Colorectal cancer	71
Breast cancer	67
Other	12
Therapy-related details	
Capecitabine monotherapy	71
Capecitabine combination therapy	79
Absolute daily dose (mg), median (range)	3000 (1000 – 5000)
Number of observed cycles per patient, mean (range)	5.2 (1 – 6)
Number of patients with treatment interruptions	33
Duration of treatment interruptions (days), median (range)	8 (1 – 118)
Number of treatment discontinuations	56
Number of observed transitions between adverse event grades	
0 → 0	254
0 → 1	93
0 → 2	41
0 → 3	7
1 → 0	26
1 → 1	125
1 → 2	44
1 → 3	9
2 → 0	8
2 → 1	34
2 → 2	69
2 → 3	12
3 → 0	2
3 → 1	6
3 → 2	9
3 → 3	22

Occurrence and severity of HFS were assessed by the patients using a questionnaire developed at the Department of Clinical Pharmacy at the University of Bonn. The description of HFS severity grades (0 to 3) was based on the descriptions provided by the CTCAE grades, version 3.0 [1]. Grade 0 was described as the absence of symptoms, patients with grade 1 had minimal skin alterations (e.g. redness) without any pain. Grade 2 was described as skin reactions (e.g. fissures, blisters, swelling) and/or pain without impairment of activities of daily living and patients with HFS grade 3 had severe skin reactions (e.g. peeling, blisters, bleeding) and/or severe pain, including impaired activities of daily living. Patients were asked to complete the questionnaire after each conducted cycle. Therefore, up to six HFS grade assessments per patient were collected. Before starting capecitabine treatment, patients were considered asymptomatic.

### Data analysis

This population pharmacodynamic analysis was performed using non-linear mixed effect modeling. Model parameters were estimated by the Laplacian method implemented in the software NONMEM 7.4.3 [19]. The likelihood-ratio test was used to discriminate between nested models. The inclusion of an extra parameter or covariate required a statistically significant reduction (*p* ≤ 0.01) of the objective function value (OFV) provided by NONMEM. Furthermore, visual predictive checks (VPC) assisted in model selection.

Implemented scripts in PsN (version 4.8.1) [20, 21] were also used for model development and R (version 3.5.1) [22] was used for visualization of results as well as generating random numbers for simulation analyses. Piraña (version 2.9.7) [23] served as a front interface.

### Model building

Since HFS can only be graded on a categorical scale, the probability of each grade was modeled with a proportional odds model which was extended with Markov elements. In this work, a minimal continuous-time Markov model (mCTMM) was applied to analyze the severity of HFS. The mCTMM was developed by Schindler and Karlsson and is a simplification of standard continuous-time Markov models [24]. A compartmental structure with four compartments was used, with each compartment representing one HFS severity grade (0, 1, 2, and 3) [7]. The probability of each grade was modeled as an amount in the respective compartment and described by differential equations in which solely transitions between adjacent states were considered (Eq. 1):

1 $$\begin{aligned} & \frac{{{\text{d}}P\left( 0 \right)}}{{{\text{d}}t}} = K_{10} \cdot P\left( 1 \right) - K_{01} \cdot P\left( 0 \right) \\ & \frac{{{\text{d}}P\left( 1 \right)}}{{{\text{d}}t}} = K_{01} \cdot P\left( 0 \right) + K_{21} \cdot P\left( 2 \right) - K_{10} \cdot P\left( 1 \right) - K_{12} \cdot P\left( 1 \right) \\ & \frac{{{\text{d}}P\left( 2 \right)}}{{{\text{d}}t}} = K_{12} \cdot P\left( 1 \right) + K_{32} \cdot P\left( 3 \right) - K_{21} \cdot P\left( 2 \right) - K_{23} \cdot P\left( 2 \right) \\ & \frac{{{\text{d}}P\left( 3 \right)}}{{{\text{d}}t}} = K_{23} \cdot P\left( 2 \right) - K_{32} \cdot P\left( 3 \right) \\ \end{aligned}$$

d*P*(grade)/d*t* represents the rate of change over time of the probability of experiencing grades 0, 1, 2 or 3, *P*(grade) is the probability of experiencing one of the HFS grades, *K*_grade,grade+1_ and *K*_grade,grade−1_ are transition rate constants for worsening to higher grades and for recovering to lower grades, respectively.

When an observation event occurred, the amount in the compartment corresponding to the respective severity grade was set to 1 whereas the other compartments were set to 0 before the next observation. This introduced the Markov property. Between two observations, rate constants defined the transitions of probabilities between different grades. In an mCTMM, it is assumed that the transition rate between two consecutive grades is independent of the grade resulting in fewer model parameters than in other Markov models. Only the mean equilibration time (MET) was introduced as a constant parameter characterizing the transition rates across different grades. The transition rate constants govern the rate at which the probability of the adverse event severity distributes between two observations. They were defined as functions of the MET and the probabilities of the respective severity grades [24].

The calculation of the probabilities experiencing one of the HFS grades was similar to a proportional odds model [25]. Since four different HFS grades were considered, three probabilities had to be estimated. The fourth probability was defined as 1 minus the sum of the three others. Logit transformation was conducted to express the respective probability as a value within the interval between 0 and 1 (Eq. 2):

2 $${\text{logit}}\left( {P\left( {{\text{Gr}}_{ij} \ge n} \right)} \right) = \log \left( {\frac{{\left( {P\left( {{\text{Gr}}_{ij} \ge n} \right)} \right)}}{{1 - \left( {P\left( {{\text{Gr}}_{ij} \ge n} \right)} \right)}}} \right) = \alpha_{n} + g\left( {x_{i} } \right) + \eta_{i}$$

Gr_*ij*_ is the HFS grade for the *i*th individual at the *j*th occasion. *P*(Gr_*ij*_ *≥* *n*) represents the probability that the HFS grade is greater than or equal to grade *n*. This can be also defined as the cumulative probability of grade *n*. *α*_*n*_ is the intercept on the logit scale and *g*(*x*_*i*_) represents a linear function on the logit scale which contains explanatory factors, such as drug exposure or covariates, such as age or sex. These factors are related to the probability experiencing HFS. *η*_*i*_ represents the interindividual random effect for the *i*th individual assuming a normal distribution with a mean of 0 and a variance of *ω*^2^. To ensure that the cumulative probability of the respective next higher grade is lower, the following parametrization of the logit intercept was used (Eq. 3):

3 $$\alpha_{n + 1} = \alpha_{n} + b_{n + 1}$$

The parameter *b*_*n*+1_ is negatively constrained and has to be estimated in the model.

Using the inverse logit function (also called expit function), $P\left( {{\text{Gr}}_{ij} \ge n} \right)$ can be directly calculated as follows (Eq. 4):

4 $$P\left( {{\text{Gr}}_{ij} \ge n} \right) = \frac{1}{{1 + {\text{e}}^{{-\left( {\alpha_{n} + g\left( {x_{i} } \right) + \eta_{i} } \right)}} }}$$

Additionally, an interindividual variability (IIV) as an exponential function of the MET was included.

After building the base model, the effects of dose and time on the MET and the logit intercepts were tested. Here, dose was tested as a time-varying covariate between therapy cycles. Moreover, a covariate analysis was performed. Continuous (patient’s age) as well as categorical covariates (sex, tumor entity and concomitant chemotherapy) were included based on their statistical significance of reducing the OFV, i.e. improving the model fit. For one additional parameter in the model the OFV had to decrease by at least 6.64 which corresponds to a *p* value ≤ 0.01 in the case of one degree of freedom. Additionally, adherence was tested as a covariate. It was measured using an electronic medication event monitoring system (MEMS™) [17, 18] and assessed as pooled overall adherence per patient over the course of therapy. Patients were allocated to one of three groups (Overall adherence > 100%, 90–100% or < 90%).

### Model evaluation

To assess the model fit, visual predictive checks for categorical data were used. 95% confidence intervals (CI) were generated from 1000 dataset simulations based on the observed dataset and superimposed by the observed proportions of patients experiencing the individual HFS grades over time.

In addition, model robustness as well as precision and bias of parameter estimates were evaluated by a non-parametric bootstrap analysis without stratification. Median and 95% CI of parameter estimates were derived from 1000 replicate datasets obtained from sampling individuals from the original dataset with replacement.

### Simulation study

The developed model was used to perform a simulation study based on 1000 virtual patients to assess the appropriateness of the standard dosing regimen for capecitabine monotherapy of 1250 mg/m^2^ twice daily and the proposed dose adjustments based on HFS severity according to the summary of product characteristics (SmPC) [15]. Since no information of body surface areas (BSA) of the patients from the observational studies [17, 18] was provided, random BSA values were generated using the rnorm function in R. BSA means and standard deviations were obtained from published data [26]. Two simulation approaches were performed: (1) A simulation was performed in 1000 virtual patients with the above-mentioned starting dose of 1250 mg/m^2^ for six cycles without dose adjustments. (2) A step-wise simulation was performed in the same 1000 patients with the same dose and total simulation duration as in (1). When meeting the criteria for dose adjustment according to the SmPC [15], the capecitabine dose was adjusted after each conducted cycle. To have an equal number of patients in both simulation scenarios, patients for whom a treatment discontinuation would be recommended were kept in the analysis. After adjusting the dose, the simulation of the subsequent cycle was performed. The HFS grade corresponding to the highest simulated probability was used to assess toxicity.

### Predictive performance

The ability of the model to predict individual HFS severity was assessed by a simulation of patients with the same characteristics as in the original dataset. Therefore, the included random effect parameters were estimated by a Bayesian approach up to a certain cycle. Then, the HFS severity of the subsequent cycle was simulated based on the Bayesian estimates and covariate effects. This approach was conducted for predictions of cycle 2 up to cycle 6. Since Markov models can only predict the probability for each toxicity grade but not the grade itself, the grade corresponding to the highest probability was compared to the respective observed HFS grade. All grades were allocated to one of the following two groups: The first group consisted of HFS grades ≥ 2 which were classified as clinically relevant since dose reductions or treatment interruptions are conducted at grade 2 or higher [15], the second group consisted of HFS grades 0 and 1. For the first group, a positive predictive value (PPV) was calculated. It indicated the ability of predicting clinically relevant HFS:

5 $${\text{PPV}} = \frac{{N\,{\text{true}}\,{\text{predicted}}\,{\text{events}}\,{\text{with}}\,{\text{grade}}\, \ge 2}}{{N\,{\text{total}}\,{\text{predicted}}\,{\text{events}}\,{\text{with}}\,{\text{grade}}\, \ge 2}}$$

The ability of predicting the absence of toxicity ≥ grade 2 was assessed by calculation of a negative predictive value (NPV) within the second group:

6 $${\text{NPV}} = \frac{{N\,{\text{true}}\,{\text{predicted}}\,{\text{events}}\,{\text{with}}\,{\text{grade}}\, \le 1}}{{N\,{\text{total}}\,{\text{predicted}}\,{\text{events}}\,{\text{with}}\,{\text{grade}}\, \le 1}}$$

Since patients were considered asymptomatic before starting therapy, predicted HFS grades at baseline were not included for calculation of both NPV and PPV.

## Results

### Model building

**Fig. 1:**
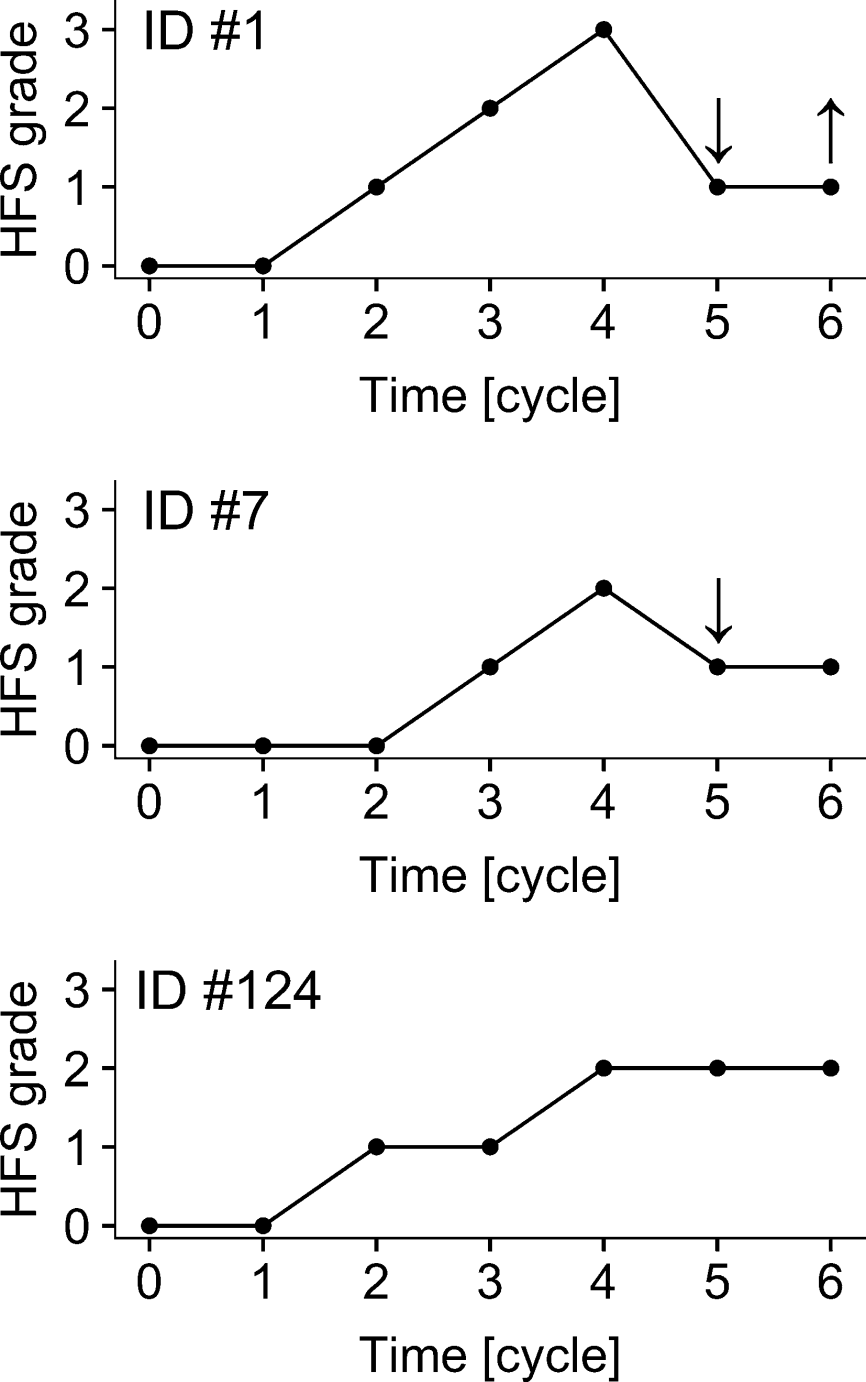
Observed hand–foot syndrome (HFS) grades over time of three representative individuals. ID #1 was a patient with a median daily starting dose of capecitabine including a dose reduction and dose increase, indicated by downwards and upwards pointing arrows, respectively. ID #7 was a patient of median age who had a dose reduction (cycle 5). ID #124 was a patient who took the median daily capecitabine dose over the whole observed period of six cycles

In total, 911 observations from 150 patients were used for model building (Table 1). Three exemplary time profiles of individual HFS severity are depicted in Fig. 1. It should be noted that 25 patients sent back HFS questionnaires after they discontinued therapy. These patients were also included in this analysis and their capecitabine dose was set to zero after discontinuation. A base minimal continuous-time Markov model (mCTMM) for hand–foot syndrome (HFS) was developed including interindividual variability (IIV) for both mean equilibration time (MET) and logit intercept *α*_*n*_, respectively. The results of the analysis of various covariates are presented in Table 2.

**Table 2:** Development of the final model including various covariates

Model	∆OFV	*p* value
Base model	0	–
Sex effect on logit intercept *α*_*n*_	− 3.454	0.063
Sex effect on MET	− 1.348	0.246
Absolute daily dose on logit intercept *α*_*n*_	− 23.45	< 0.00001
Absolute daily dose on MET	− 0.445	0.505
Capecitabine monotherapy (yes/no) on logit intercept *α*_*n*_	− 1.358	0.244
Capecitabine monotherapy (yes/no) on MET	+ 1.006	–
Breast cancer (yes/no) on logit intercept *α*_*n*_	− 1.274	0.259
Breast cancer (yes/no) on MET	− 0.139	0.709
Colorectal cancer (yes/no) on logit intercept *α*_*n*_	− 1.978	0.160
Colorectal cancer (yes/no) on MET	− 0.467	0.494
Other tumor entities (yes/no) on logit intercept *α*_*n*_	− 0.391	0.532
Other tumor entities (yes/no) on MET	+ 0.488	–
Age effect on logit intercept *α*_*n*_	− 0.077	0.930
Age effect on MET	− 0.132	0.716
Overall adherence (> 100%/90–100%/ < 90%) on logit intercept *α*_*n*_	− 0.130	0.937^a^
Overall adherence (> 100%/90–100%/< 90% adherence) on MET	− 1.316	0.518^a^
Time effect on logit intercept *α*_*n*_	− 4.179	0.041
Time effect on MET	− 1.4	0.237

*∆OFV* difference in the objective function value between the covariate model and the base model, *MET* mean equilibration time

^a^Two degrees of freedom

The final mCTMM included a linear effect of absolute daily capecitabine dose on the logit intercept *α*_*n*_ indicating larger probabilities of experiencing HFS with an increasing dose (∆OFV = − 23.45, *p* < 0.00001). None of the other examined covariates or time effects resulted in a statistically significant reduction of the OFV after inclusion. Additionally, after the dose effect was included into the base model, a further analysis of the mentioned covariates or time effects did not result in a significant improvement of the model fit. The final equation described the cumulative probabilities as follows (Eq. 7):

7 $$P\left( {{\text{Gr}}_{ij} \ge n} \right) = \frac{1}{{1 + {\text{e}}^ {{ - \left( {\alpha_{n} + \theta_{{{\text{Dose}}}} \times \left( {{\text{Dose (mg)}} - 3000\,{\text{mg}}} \right) + \eta_{i} } \right)}} }}$$

*Θ*_Dose_ represents the slope of the linear dose effect on the logit scale. The dose effect was centered on the population median daily dose of 3000 mg. The final model code used in NONMEM is provided in the electronic supplementary material.

A summary of the parameter estimates including the bootstrap results is depicted in Table 3. Parameters were well estimated with relative standard errors below 25%, except for the IIV parameter associated with *α*_1_.

**Table 3:** Parameter estimates

Parameter	Estimate (relative standard error, %)	Bootstrap median	Bootstrap 95% confidence intervals
*α*_1_	1.81 (14)	1.88	1.38 to 2.51
*b*_2_	− 1.80 (11)	− 1.79	− 2.23 to (− 1.45)
*b*_3_	− 2.08 (13)	− 2.05	− 2.73 to (− 1.57)
MET (cycle)	1.09 (10)	1.11	0.896 to 1.430
*Θ*_Dose_	8.33 × 10^–4^ (24)	8.28 × 10^–4^	4.05 × 10^–4^ to 1.48 × 10^–3^
*ω*_*α*1_	1.12 (37)	0.981	0.0112 to 1.65
*ω*_MET_	0.542 (22)	0.560	0.310 to 0.842

*α1* intercept parameter on the logit scale for HFS grade 1, *bn* parameter for grade *n* such that *αn* = *αn-1* + *bn*, *MET* mean equilibration time, *Θ*_*Dose*_ slope of the linear daily dose effect on the logit scale, *ωP* standard deviation of the interindividual variability of parameter *P*

### Model evaluation

**Fig. 2:**
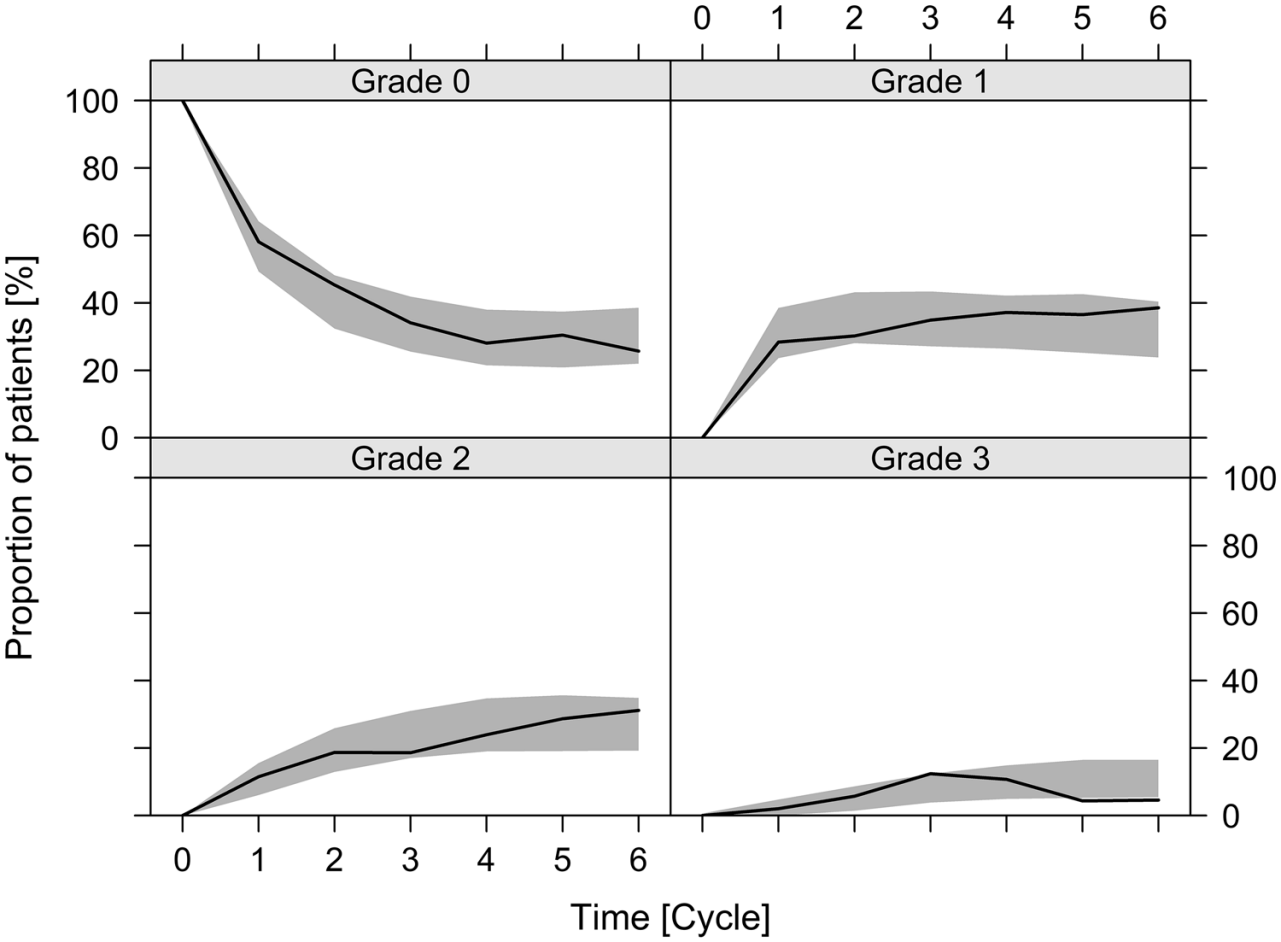
Categorical visual predictive check showing the proportions of patients experiencing patient-reported CTCAE-based HFS grades from 0 to 3 over time. Solid black lines indicate the observed proportion of patients and the grey shaded areas are the 95% confidence intervals of simulated proportions based on 1000 simulated datasets using the final model

The categorical visual predictive check revealed an accurate description of the provided data. The simulated proportions of patients experiencing one of the HFS grades described the respective observed proportions of patients over time well (Fig. 2).

### Simulation study

**Fig. 3:**
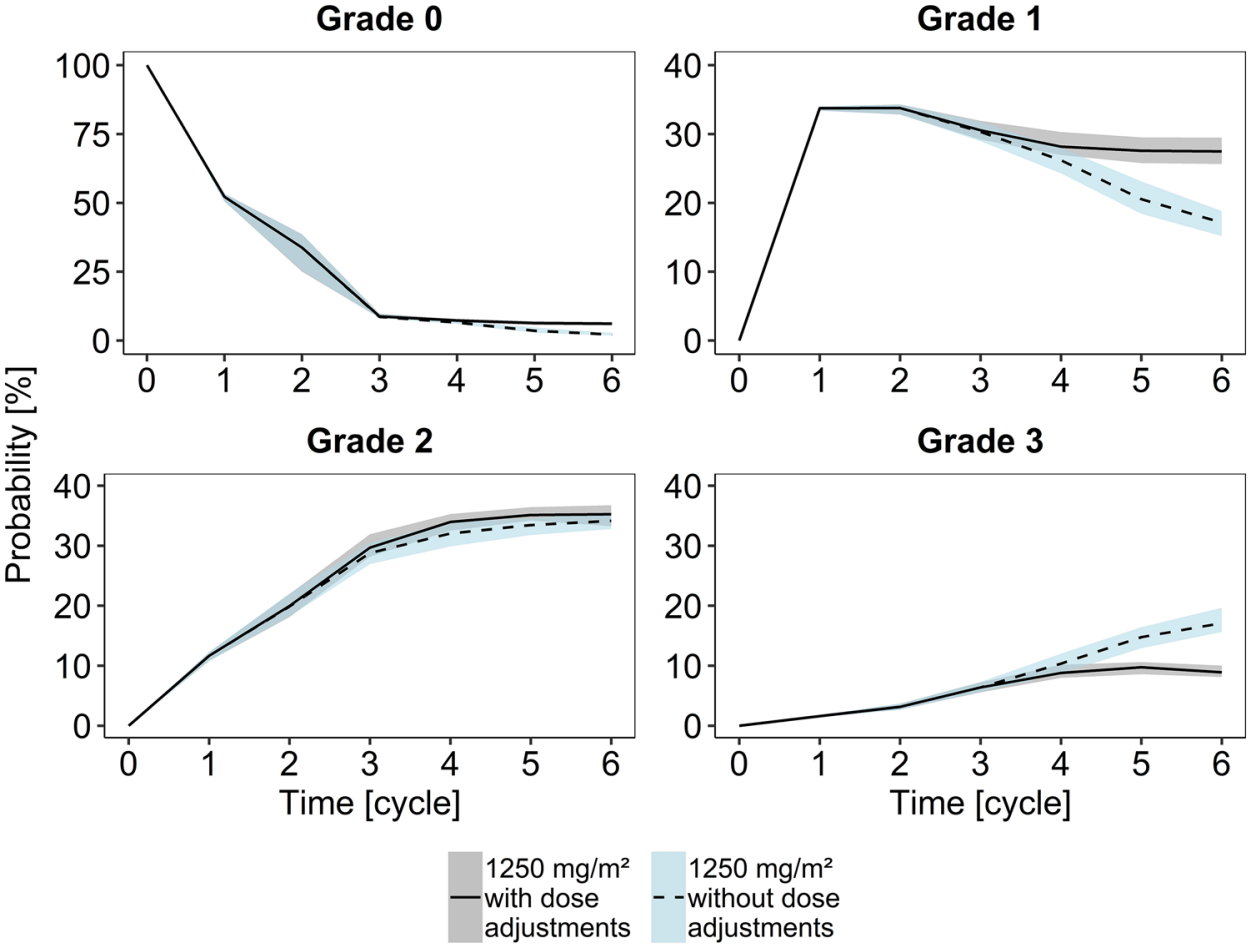
Simulated probabilities versus time for HFS grades 0–3 of 1000 virtual patients. Solid lines indicate the median probability when dose adjustments were performed according to the capecitabine SmPC [15]. Grey shaded areas are the respective 95% confidence intervals of the median. Dashed lines indicate the median probability when no dose adjustments were performed. Blue shaded areas are the respective 95% confidence intervals of the median

Based on the developed dose–toxicity model and on the results of the simulation study (Fig. 3), it was evident that dose adjustments decreased the probability of severe HFS during therapy while increasing the probabilities of the absence of clinically relevant toxicity (grades 0 and 1). In particular, grade 3 toxicity was more probable when no dose adjustments were performed whereas the probabilities of grade 2 did not differ between the two simulation approaches. The simulation study also clearly showed that patients without dose adjustments tended to remain in grade 3 for a longer period of time which is characterized by a higher transition count from grade 3 to 3 compared to the approach which included dose adjustments (transition count of 442 and 234, respectively). However, the transition counts from grade 2 to 2 were comparable in both simulation groups (769 without and 774 with dose adjustments).

### Predictive performance

The predictive ability of the model for individual patients was assessed by calculating the positive and negative predictive value (PPV, NPV) for each cycle (from cycle 2) based on Bayesian estimates of both random effect parameters from the previous cycle as well as the dose effect. PPV ranged from 21.9 to 34.2% whereas NPV ranged from 61.9 to 73.3%. Both values indicated a rather poor predictive performance on an individual patient level.

## Discussion

This is the first study evaluating the time course of patient-reported adverse event severity in clinical routine during anti-cancer therapy with a Markov modeling approach. A heterogeneous patient group with different tumor entities was analyzed regarding the occurrence and severity of HFS during treatment with capecitabine. A parsimonious version of a continuous-time Markov model, the mCTMM, was applied requiring fewer model parameters to be estimated compared to other Markov modeling approaches [24]. Thus, the mCTMM can also be applied to sparse-data situations to obtain precisely estimated parameters. Additionally, only transitions between adjacent grades were allowed since only a small proportion of transitions between non-neighboring grades were observed (Table 1). Therefore and because of the generally small number of observations per patient, a mCTMM was chosen instead of a continuous-time Markov model. The absolute daily dose of capecitabine was found to be a predictor of development of HFS which was in accordance with the observed dose-dependency [14, 27]. Since data on height and weight were not gathered in the studies used for this model, effects of normalized doses could not be investigated. Other covariates did not lead to a significant model improvement including overall adherence. A previous study found a possible influence of over-adherence on high-grade toxicity [28]. In addition, the study of Hénin et al. in which clinician-reported HFS severity in patients with colorectal cancer was analyzed with a discrete-time Markov model, found that creatinine clearance was a significant covariate for HFS severity [16]. However, renal function was not estimated in both studies used for our model. Therefore, a wider selection of covariates would potentially be able to improve the model fit.

Model parameters could be well estimated except for a comparatively higher standard error of the IIV of *α*_1_. This phenomenon was also observed by Schindler and Karlsson [24]. They suggested that the absence of HFS before starting therapy (at therapy cycle zero) caused these large uncertainties of IIV [24]. However, the logit intercept parameter itself could be precisely estimated in this study. Another reason for larger uncertainties of the IIV parameter estimate may be due to the overall low number of transitions between HFS grades per patient. Only a maximum of seven time points could be analyzed (one per therapy cycle plus baseline grade) in which the patients reported the maximum HFS grade per therapy cycle. Therefore, distinguishing between HFS severities within the respective cycle was not possible which resulted in a low transition number. For the same reason, time delays due to treatment interruptions could not be considered for this model. The time variation of covariates within one cycle (such as dose) could not be implemented either. A more frequent grading would be required to improve the ability of Markov models of predicting the probabilities of the respective grades for individual patients as shown in the study of Lu et al. [29]. For example, an already validated one-week recall period as in the PRO-CTCAE item library [30, 31] would be more suitable for model development. However, our questionnaire was developed before a German version of the PRO-CTCAE questionnaire was available [32]. Using a validated, entity-specific PRO-CTCAE questionnaire would enhance the development and application of Markov models for evaluation of categorical adverse event severity.

Despite the subjectivity of the patient-reported HFS severity, the limited number of both observed grades and potential covariates as well as the real-world setting, the model was able to accurately describe the observed data on the population level. It also showed that the recommended dosage regimen of 1250 mg/m^2^ for capecitabine monotherapy is appropriate to minimize the probability of HFS grade 3 and increase the probability of the absence of clinically relevant toxicity. Thus, population-based recommendations of dose adjustments can be supported using this model. However, the predictive performance for individual patients was not satisfactory which is probably due to the limited number of observations. As mentioned above, a more frequent grading, particularly within a therapy cycle, might enhance the individual predictive performance. Another reason could be the assumption that the patient-reported HFS grade equaled the “true” grade. Therefore, a misclassification of the actual grade could not be excluded. A possibility to account for the error between a categorical observation and the actual grade would be a model extension towards a hidden Markov model [33]. In such a model the unobserved “true” grade could be described as well. Therefore, our model has to be further improved before it can be applied to make individual predictions.

In conclusion, minimal continuous-time Markov models can be set up using patient-reported outcomes. Our modeling framework may assist in the optimization of dosage regimens and adjustment strategies on the population level aiming at minimizing symptom burden during anti-cancer drug therapy. Predictive performance on the individual patient level may be improved by more frequent PRO measurements and more sophisticated modeling approaches.

## Electronic supplementary material

Below is the link to the electronic supplementary material.

Supplementary file1 (PDF 951 kb)

## Data Availability

Data are available from the corresponding author upon reasonable request.

## References

[1] National Cancer Institute (2018) Common terminology criteria for adverse events v5.0 (CTCAE). https://ctep.cancer.gov. Accessed 20 Aug 2020

[2] Basch E (2009). Patient-reported outcomes in drug safety evaluation. Ann Oncol.

[3] Galizia D, Milani A, Geuna E, Martinello R, Cagnazzo C, Foresto M (2018). Self-evaluation of duration of adjuvant chemotherapy side effects in breast cancer patients: a prospective study. Cancer Med.

[4] Basch E, Reeve BB, Mitchell SA, Clauser SB, Minasian LM, Dueck AC (2014). Development of the National Cancer Institute’s patient-reported outcomes version of the common terminology criteria for adverse events (PRO-CTCAE). J Natl Cancer Inst.

[5] Friberg LE, Henningsson A, Maas H, Nguyen L, Karlsson MO (2002). Model of chemotherapy-induced myelosuppression with parameter consistency across drugs. J Clin Oncol.

[6] Karlsson MO, Schoemaker RC, Kemp B, Cohen AF, van Gerven JM, Tuk B (2000). A pharmacodynamic Markov mixed-effects model for the effect of temazepam on sleep. Clin Pharmacol Ther.

[7] Suleiman AA, Frechen S, Scheffler M, Zander T, Nogova L, Kocher M (2015). A modeling and simulation framework for adverse events in erlotinib-treated non-small-cell lung cancer patients. AAPS J.

[8] Niebecker R, Maas H, Staab A, Freiwald M, Karlsson MO (2019). Modeling exposure-driven adverse event time courses in oncology exemplified by afatinib. CPT Pharmacomet Syst Pharmacol.

[9] Keizer RJ, Gupta A, Mac Gillavry MR, Jansen M, Wanders J, Beijnen JH (2010). A model of hypertension and proteinuria in cancer patients treated with the anti-angiogenic drug E7080. J Pharmacokinet Pharmacodyn.

[10] Lacroix BD, Karlsson MO, Friberg LE (2014). Simultaneous exposure-response modeling of ACR20, ACR50, and ACR70 improvement scores in rheumatoid arthritis patients treated with certolizumab pegol. CPT Pharmacomet Syst Pharmacol.

[11] Reigner B, Blesch K, Weidekamm E (2001). Clinical pharmacokinetics of capecitabine. Clin Pharmacokinet.

[12] Cassidy J, Twelves C, van Cutsem E, Hoff P, Bajetta E, Boyer M (2002). First-line oral capecitabine therapy in metastatic colorectal cancer: a favorable safety profile compared with intravenous 5-fluorouracil/leucovorin. Ann Oncol.

[13] Twelves C, Scheithauer W, McKendrick J, Seitz JF, van Hazel G, Wong A (2012). Capecitabine versus 5-fluorouracil/folinic acid as adjuvant therapy for stage III colon cancer: Final results from the X-ACT trial with analysis by age and preliminary evidence of a pharmacodynamic marker of efficacy. Ann Oncol.

[14] Scheithauer W, Blum J (2004). Coming to grips with hand-foot syndrome. Insights from clinical trials evaluating capecitabine. Oncology (Williston Park, NY).

[15] Electronic Medicines Compendium (2018) Capecitabine 500 mg film-coated tablets: summary of product characteristics. https://www.medicines.org.uk/emc/product/9939. Accessed 20 Aug 2020

[16] Hénin E, You B, VanCutsem E, Hoff PM, Cassidy J, Twelves C (2009). A dynamic model of hand-and-foot syndrome in patients receiving capecitabine. Clin Pharmacol Ther.

[17] Simons S, Ringsdorf S, Braun M, Mey UJ, Schwindt PF, Ko YD (2011). Enhancing adherence to capecitabine chemotherapy by means of multidisciplinary pharmaceutical care. Support Care Cancer.

[18] Krolop L, Ko YD, Schwindt PF, Schumacher C, Fimmers R, Jaehde U (2013). Adherence management for patients with cancer taking capecitabine: a prospective two-arm cohort study. BMJ Open.

[19] Beal S, Sheiner LB, Boeckmann A, Bauer RJ (2018). NONMEM user's guides (1989–2018).

[20] Lindbom L, Pihlgren P, Jonsson EN, Jonsson N (2005). PsN-Toolkit—a collection of computer intensive statistical methods for non-linear mixed effect modeling using NONMEM. Comput Methods Programs Biomed.

[21] Lindbom L, Ribbing J, Jonsson EN (2004). Perl-speaks-NONMEM (PsN)—a Perl module for NONMEM related programming. Comput Methods Programs Biomed.

[22] R Core Team (2018) R: a language and environment for statistical computing. R Foundation for Statistical Computing, Vienna, Austria. https://www.R-project.org/. Accessed 11 Mar 2020

[23] Keizer RJ, Karlsson MO, Hooker A (2013). Modeling and simulation workbench for NONMEM: tutorial on Pirana, PsN, and Xpose. CPT Pharmacomet Syst Pharmacol.

[24] Schindler E, Karlsson MO (2017). A minimal continuous-time Markov pharmacometric model. AAPS J.

[25] Sheiner LB (1994). A new approach to the analysis of analgesic drug trials, illustrated with bromfenac data. Clin Pharmacol Ther.

[26] de Man FM, Veerman GDM, Oomen-de Hoop E, Deenen MJ, Meulendijks D, Mandigers CMPW (2019). Comparison of toxicity and effectiveness between fixed-dose and body surface area-based dose capecitabine. Ther Adv Med Oncol.

[27] Kara IO, Sahin B, Erkisi M (2006). Palmar-plantar erythrodysesthesia due to docetaxel-capecitabine therapy is treated with vitamin E without dose reduction. Breast.

[28] Le Saux O, Bourmaud A, Rioufo C, Colomban O, Guitton J, Schwiertz V (2018). Over-adherence to capecitabine: a potential safety issue in breast and colorectal cancer patients. Cancer Chemother Pharmacol.

[29] Lu T, Yang Y, Jin JY, Kågedal M (2020). Analysis of Longitudinal ordered categorical data for muscle spasm adverse event of vismodegib: comparison between different pharmacometric models. CPT Pharmacomet Syst Pharmacol.

[30] Mendoza TR, Dueck AC, Bennett AV, Mitchell SA, Reeve BB, Atkinson TM (2017). Evaluation of different recall periods for the US National Cancer Institute's PRO-CTCAE. Clin Trials.

[31] Dueck AC, Mendoza TR, Mitchell SA, Reeve BB, Castro KM, Rogak LJ (2015). Validity and reliability of the US National Cancer Institute's Patient-reported outcomes version of the common terminology criteria for adverse events (PRO-CTCAE). JAMA Oncol.

[32] Hagelstein V, Ortland I, Wilmer A, Mitchell SA, Jaehde U (2016). Validation of the German patient-reported outcomes version of the common terminology criteria for adverse events (PRO-CTCAE™). Ann Oncol.

[33] Brekkan A, Jönsson S, Karlsson MO, Plan EL (2019). Handling underlying discrete variables with bivariate mixed hidden markov models in NONMEM. J Pharmacokinet Pharmacodyn.

