# Supplementary material for: Evaluation of patient-reported severity of hand–foot syndrome under capecitabine using a Markov modeling approach
Source: Cancer Chemother Pharmacol. 2020 Aug 27;86(3):435–44. doi: 10.1007/s00280-020-04128-7 (PMC7478943; doi:10.1007/s00280-020-04128-7)
Supplement: Supplementary file 1 — Supplementary file1 (PDF 951 kb) [file 280_2020_4128_MOESM1_ESM.pdf]

Supplementary Document

Evaluation of patient-reported severity of hand-foot syndrome under capecitabine using a Markov modeling approach

Eduard Schmulenson[1], Linda Krolop[1], Sven Simons[1], Susanne Ringsdorf[1], Yon-Dschun Ko[2], Ulrich Jaehde[1]*

[1]Institute of Pharmacy, Department of Clinical Pharmacy, University of Bonn, Bonn, Germany

[2]Department of Internal Medicine, Evangelische Kliniken Bonn gGmbH, Johanniter Hospital, Bonn, Germany

***Corresponding author**

Prof. Dr. Ulrich Jaehde

Institute of Pharmacy

University of Bonn

An der Immenburg 4

53121 Bonn

Germany

Phone: +49 228 73 5252

Fax: +49 228 73 9757

Email: u.jaehde@uni-bonn.de

NONMEM code of the final model

```
$PROBLEM Capecitabin HFS mCTMM

$INPUT

ID ; No. of the patient

TIME ; Therapy cycle no. as time unit

DV ; No AE -> DV = 1, otherwise DV = 2-4 (grades 1-3)

AMT ; Probability for AE compartments

CMT ; 1-4 = AE compartments

DOSEDAY ; Absolute dose per day

EVID

MDV

AGE

SEX

MAMMA ; tumor entity: mamma carcinoma

COLON ; tumor entity: colorectal cancer

OTHER ; other tumor entity

MONO ; Capecitabin monotherapy yes/no


$DATA HFS_total.csv

$SUBROUTINES ADVAN6 TOL = 4

$MODEL

NCOMP = 4

COMP = (G0) ; No AE

COMP = (G1) ; Mild AE

COMP = (G2) ; Moderate AE

COMP = (G3) ; Severe AE
```

```
$ABB COMRES = 1

$PK

IF(NEWIND.NE.2) THEN

PSDV = 0

COM(1) = 0

ENDIF

PRSP = PSDV ; Previous DV

IF(TIME.EQ.0) F1 = 1

IF(PRSP.EQ.1) COM(1) = 0

IF(PRSP.EQ.2) COM(1) = 1

IF(PRSP.EQ.3) COM(1) = 2

IF(PRSP.EQ.4) COM(1) = 3


F1=0

F2=0

F3=0

F4=0


IF(COM(1).EQ.0) F1 = 1

IF(COM(1).EQ.1) F2 = 1

IF(COM(1).EQ.2) F3 = 1

IF(COM(1).EQ.3) F4 = 1


;--- mCTMM model parameters

MET = THETA(4)*EXP(ETA(2))    ; Mean equilibration time

ET1 = ETA(1)
```

```
$DES

A1 = THETA(1)+ET1 + THETA(5)*(DOSEDAY-3000)            ; alpha_1 with linear dose effect

B2 = THETA(2)                 ; beta_2

B3 = THETA(3)              ; beta_3


; Logit of the cumulative probabilities

LGE1 = A1                    ; >=Grade 1

LGE2 = LGE1 + B2                  ; >=Grade 2

LGE3 = B3 + LGE2              ; Grade 3


; Cumulative probabilities

PGE1 = EXP(LGE1)/(1+EXP(LGE1))    ; >=Grade 1

PGE2 = EXP(LGE2)/(1+EXP(LGE2))    ; >=Grade 2

PGE3 = EXP(LGE3)/(1+EXP(LGE3))    ; Grade 3


; Probabilities

P0   = (1-PGE1)                 ; Probability of grade 0

P1   = (PGE1-PGE2)              ; Probability of grade 1

P2   = (PGE2-PGE3)              ; Probability of grade 2

P3   = PGE3                    ; Probability of grade 3


; Transfer rate constants

L01 = 1/(MET*(1+P0/P1))

L10 = L01 *P0/P1

L12 = 1/(MET*(1+P1/P2))

L21 = L12 *P1/P2

L23 = 1/(MET*(1+P2/P3))

L32 = L23 *P2/P3
```

```
; Differential equations for the probability of each grade

DADT(1) = L10*A(2)         - L01     *A(1) ; Grade 0

DADT(2) = L01*A(1)+L21*A(3)-(L10+L12)*A(2) ; Grade 1

DADT(3) = L12*A(2)+L32*A(4)-(L21+L23)*A(3) ; Grade 2

DADT(4) = L23*A(3)         - L32     *A(4) ; Grade 3


$ERROR

;--- Redefine variables from $DES

A1X = THETA(1) + ET1+ THETA(5)*(DOSEDAY-3000)

B2X = THETA(2)

B3X = THETA(3)


LGE1X = A1X

LGE2X = LGE1X + B2X

LGE3X = B3X + LGE2X


PGE1X = EXP(LGE1X)/(1+EXP(LGE1X))

PGE2X = EXP(LGE2X)/(1+EXP(LGE2X))

PGE3X = EXP(LGE3X)/(1+EXP(LGE3X))


P0X = (1-PGE1X)

P1X = (PGE1X-PGE2X)

P2X = (PGE2X-PGE3X)

P3X = PGE3X


; --- Define Y

P0X = A(1) ; Probability of observing grade 0

P1X = A(2) ; Probability of observing grade 1
```

```
P2X = A(3) ; Probability of observing grade 2

P3X = A(4) ; Probability of observing grade 3


IF(DV.EQ.1.AND.CMT.EQ.0) Y=P0X

IF(DV.EQ.2.AND.CMT.EQ.0) Y=P1X

IF(DV.EQ.3.AND.CMT.EQ.0) Y=P2X

IF(DV.EQ.4.AND.CMT.EQ.0) Y=P3X


; Cumulative probabilities

CUP0 = P0X

CUP1 = CUP0 + P1X

CUP2 = CUP1 + P2X

CUP3 = CUP2 + P3X


; Start of simulation

IF(ICALL.EQ.4) THEN

IF(CMT.EQ.0) THEN

  CALL RANDOM (2,R)

     IF(R.LE.CUP0) DV = 1

     IF(R.GT.CUP0.AND.R.LE.CUP1) DV = 2

     IF(R.GT.CUP1.AND.R.LE.CUP2) DV = 3

     IF(R.GT.CUP2) DV = 4

ENDIF

ENDIF

; End of simulation

; Store DV

PSDV = DV
```

```
$THETA

(1.81) ; 1 alpha_1

(-1000000, -1.8,0) ; 2 beta_2

(-1000000, -2.08,0) ; 3 beta_3

(0, 1.09) ; 4 MET

(0.00083) ; Dose effect


$OMEGA

1.25 ; IIV alpha_1

0.294 ; IIV MET


$COV PRINT=E MATRIX=R

$ESTIMATION MAXEVAL=9999 METHOD=1 LAPLACE LIKE PRINT=1 NOABORT SIG=2

;$SIM (7776) (8877 UNIFORM) ONLYSIM NOPREDICTION
```
